# Relationship between environmental and sustainability-related literacy and health behaviors: a systematic review

**DOI:** 10.3389/fpubh.2026.1860395

**Published:** 2026-07-27

**Authors:** L. Paulsen, H. Muth, J. Kallenbach, L. Reismann, C. Jochem, J. Bucksch

**Affiliations:** 1 Prevention and Health Promotion, Faculty of Natural and Sociological Sciences, Heidelberg University of Education, Heidelberg, Germany; 2 Chair of Planetary & Public Health, Faculty of Law, Business & Economics, University of Bayreuth, Bayreuth, Germany; 3 Department of Education, Faculty of Human Sciences, MSH Medical School Hamburg, University of Applied Sciences and Medical University, Hamburg, Germany

**Keywords:** environment, health behavior, healthy lifestyle, literacy, sustainability

## Abstract

**Introduction:**

The climate crisis poses significant risks to environmental and human health, emphasizing the need for integrated approaches such as planetary health and related co-benefits. In this context, environmentally sustainable and health-promoting behaviors are often closely aligned, suggesting that environmental and sustainability-related literacy may positively influence health behaviors. However, this association has not been systematically synthesized. Therefore, this review aimed to examine the relationship between environmental and sustainability-related literacy and health behaviors.

**Methods:**

Following PRISMA guidelines and a preregistered protocol (PROSPERO CRD420251104050), literature searches were conducted in seven databases on 9 April 2025. Studies examining the association between environmental and sustainability-related literacy and health behaviors were included. Screening, data extraction, and quality appraisal (using the Joanna Briggs Institute checklist) were conducted independently by two reviewers. Due to heterogeneity in concepts and measures, a narrative synthesis was performed.

**Results:**

Of 7,864 non-duplicate records, five cross-sectional studies (2020–2024) were included. One study identified an explanatory pathway linking environmental literacy to health behaviors via environmental attitudes and sustainable lifestyles. Three studies reported positive associations between environmental and sustainability-related literacy and health behaviors, including diet and smoking. One study did not report results for the specific association examined in this review. Overall study quality was mixed.

**Discussion:**

Findings suggest a potential positive relationship between environmental and sustainability-related literacy and health behaviors, supporting the idea of co-benefits between sustainability and health. However, the small number of studies, their cross-sectional design, and conceptual and methodological heterogeneity restrict firm conclusions. Further research using robust designs, clearer conceptual frameworks, and standardized measures is needed to better understand causal relationships. Strengthening environmental and sustainability-related literacy could support strategies to promote both public health and environmental sustainability, alongside necessary structural and policy-level actions.

**Systematic review registration:**

PROSPERO, CRD420251104050.

## 1 Introduction

Currently, the climate crisis represents one of the most pressing global challenges, with far-reaching consequences for ecosystems and societies (1–3). Beyond environmental degradation, a wide range of climate-related phenomena pose substantial risks to human health, including both direct (e.g., heat-related illness and injuries from extreme weather events) and indirect (e.g., on mental health such as chronic stress or anxiety) effects (4–7). In response to these intertwined challenges, the concept of planetary health has emerged, emphasizing the complex interconnections between human health and the social, political, and economic systems, as well as the planet’s natural systems. It calls for transformative action to preserve the natural foundations of life for present and future generations (8–10).

Addressing the climate crisis, particularly through the reduction of greenhouse gas emissions, requires structural and policy-level interventions as well as meaningful changes in individual behavior (11–13). In this context, environmental and sustainability-related literacy plays a crucial role as a key enabling factor. It equips individuals with the knowledge, skills, awareness, and sense of responsibility needed to understand environmental challenges and to adopt pro-environmental and ecological behaviors (14–20).

The planetary health approach thereby highlights the interconnectedness of health and earth’s natural systems, for example in health co-benefits, referring to positive health outcomes that arise from climate change mitigation strategies (21, 22). For example, adopting an active travel lifestyle, such as cycling or walking, can simultaneously reduce greenhouse gas emissions and increase physical activity, thereby improving health (23–25). These synergies illustrate that environmentally sustainable behaviors and health-promoting behaviors are often closely aligned, suggesting that fostering environmental and sustainability-related literacy may not only support ecological outcomes but could also positively influence health behaviors (20).

Building on this perspective, previous studies point to a potential transfer effect, whereby environmental and sustainability-related literacy may extend beyond ecological behavior and also shape health-related behavior. For instance, environmental awareness and connectedness to nature have been linked to greater engagement in active and ecological lifestyles, including cycling and walking, which support both health and emission reduction (26, 27).

Despite these promising indications, the relationship between environmental and sustainability-related literacy and health behaviors has not yet been systematically synthesized. While prior research, including a scoping review, has examined links between environmental behaviors and health behaviors (e.g., active travel and physical activity or recycling and sedentary behavior), the specific role of environmental and sustainability-related literacy remains scarce (28).

Therefore, this systematic review aims to synthesize current findings on the association between environmental and sustainability-related literacy and various forms of health behaviors, exploring, e.g., whether individuals with higher levels of environmental literacy—such as those actively engaged in climate protection—are more likely to adopt healthier behaviors. Synthesizing these findings will contribute to the scientific discourse, identify gaps in the literature, and inform future interventions or policies aimed at promoting both environmental sustainability and public health.

## 2 Methods

The present systematic review adhered to the Preferred Reporting Items for Systematic Reviews and Meta-Analyses (PRISMA) guidelines (29–31) and was registered in the PROSPERO database (CRD420251104050).

### 2.1 Data sources, search strategy and study selection

On April 9, 2025, systematic literature searches were conducted in MEDLINE (via PubMed and OVID), Scopus, Web of Science, APA PsycINFO, PSYNDEX Literature with PSYNDEX Tests, and GreenFILE to identify studies investigating the relationship between environmental and sustainability-related literacy and health behaviors. These databases were selected to encompass a wide range of disciplines in the fields of public health, health promotion, and environmental research.

Our search strategies across all seven databases combined keywords related to environmental and sustainability-related literacy (e.g., “green literacy,” “climate literacy”) and health behaviors. Health behaviors refer to actions undertaken by individuals that influence health, morbidity, and mortality—both positively and negatively—within a society (20, 32–34). These behaviors encompass a wide range of domains, including smoking, substance use, dietary practices, physical activity, and sleep (20, 32, 33). Additional potential keywords (e.g., socially responsible, ethical consum*, resilient, mindful, nature-based, low impact, resource-efficient, zero waste, energy-efficient) were excluded based on a sensitivity analysis conducted in PubMed, as they did not yield any additional relevant records. The full search strategies for each database are provided in the Supplementary material 1. In addition, we conducted supplementary searches in Google Scholar, screened the reference lists of relevant studies (26, 28), and performed manual searches. We also utilized AI-based tools such as SciSpace and Elicit to identify further relevant literature. These AI-based tools were used in a supplementary, exploratory capacity only, to help confirm the comprehensiveness of the systematic database searches, and not as a primary search method. Any additional records identified through these tools were subjected to the same predefined eligibility criteria and dual independent screening process described below; no study was included on the basis of AI-generated output alone.

All search results were imported into the reference management software Citavi, subsequently exported as RIS files and uploaded to the web-based systematic review management platform Cadima (version 2.2.4.2), which supported the screening process of this review by enabling the automated removal of duplicate records and a structured two-step screening procedure at both the title/abstract and full-text levels using predefined eligibility criteria. All duplicates were removed prior to screening, resulting in 7,864 non-duplicate records. After a consistency check with 20 studies, two reviewers out of a team of reviewers (LP, JB, CJ, LR, JK, HM) independently screened the titles and abstracts of all 7,864 records against the predefined eligibility criteria. Any disagreements were resolved through discussion involving a third or fourth reviewer (LP, JB). Full-text articles were retrieved when the information in the title and abstract met the inclusion criteria or when eligibility remained uncertain. Two reviewers then independently screened all 122 full-texts of potentially relevant records in their entirety, with conflicts again resolved by consultation with a third or fourth reviewer (LP, JB). Data extraction and quality assessment were conducted outside of Cadima, using Microsoft Excel and Microsoft Word, respectively (see below).

### 2.2 Eligibility criteria

We included empirical studies employing quantitative, qualitative, or mixed-methods approaches that were published as peer-reviewed journal articles. All other publication types (e.g., editorials, study protocols, commentaries, and conference abstracts) were excluded. No restrictions were applied regarding language or publication date. Additional eligibility criteria were developed based on the PEO framework (Population, Exposure and Outcome; see Table 1).

**Table 1:** Eligibility criteria based on the PEO framework.

Eligibility criteria	
Population (P)	
Inclusion criteria: individuals of any age group (e.g., children, adolescents, and adults) from any geographical or cultural context; capable of making conscious decisions about their own behavior	Exclusion criteria: change agents, multipliers, or stakeholders who influence or make decisions on behalf of individuals or target groups
Exposure (E)	
Inclusion criteria: studies that assess environmental and sustainability-related literacy (e.g., also climate literacy, green literacy, sustainable food literacy), including its components such as competencies measurement instruments and timing of assessment could vary	Exclusion criteria: studies that did not assess the influence of environmental or sustainability-related literacy studies focused solely on environmental or sustainability-related literacy without reference to health behaviors studies investigated toxic, chemical, or physical environmental influences on health studies addressing only environmental health literacy or health literacy
Outcome (O)	
Inclusion criteria: studies measuring health behaviors such as physical activity, healthy eating, or substance use (e.g., alcohol, tobacco) measurement instruments and timing of assessment could vary	Exclusion criteria: studies that did not assess health behaviors that reported only health outcomes (e.g., BMI)

### 2.3 Data extraction and quality assessment

A standardized data extraction form was developed in Microsoft Excel and was piloted on one included study by two reviewers to ensure consistency and clarity (LP, HM). Subsequently, the two reviewers (LP, HM) independently extracted data from all eligible studies. Any disagreements were resolved through discussion, involving a third independent reviewer (JB, CJ) when necessary.

The following information was extracted from each study: author(s), publication year, study title, data location, aim of the study, study design, sample characteristics (e.g., target group, sample size, age range, mean age, gender distribution), conceptual model, understanding and measure of environmental and sustainability-related literacy, understanding and measure of health behaviors, as well as reported associations between environmental and sustainability-related literacy and health behaviors. If information was missing, the authors were contacted via e-mail.

The methodological quality and risk of bias of all included studies were independently assessed by two reviewers (LP, JB) using the Joanna Briggs Institute (JBI) Critical Appraisal Checklist for cross-sectional studies (35, 36), which was documented in Microsoft Word, as only cross-sectional studies were included in the final analysis. The checklist consists of eight items with the response options “yes,” “no,” “unclear,” and “not applicable.” It covers key methodological aspects, including sampling procedures, the validity and reliability of the exposure measures, the control of confounding variables, the validity and reliability of the outcome measures, and the appropriateness of the statistical approaches used (35, 36). Any disagreements between the reviewers regarding the quality assessment of individual studies were resolved through discussion, involving a third reviewer (CJ) when necessary. No studies were excluded from the review on the basis of their methodological quality.

### 2.4 Data synthesis and analysis

The characteristics of the included studies were summarized descriptively; the findings were synthesized narratively, with a focus on identifying consistent patterns, variations across studies, and potential explanatory factors, while taking into account study quality and methodological diversity.

Due to substantial heterogeneity in the conceptualization and measurement of environmental and sustainability-related literacy, as well as in the measures and operationalization of health behaviors, statistical pooling of results was not feasible. Therefore, a meta-analysis was not conducted.

## 3 Results

**Figure 1:**
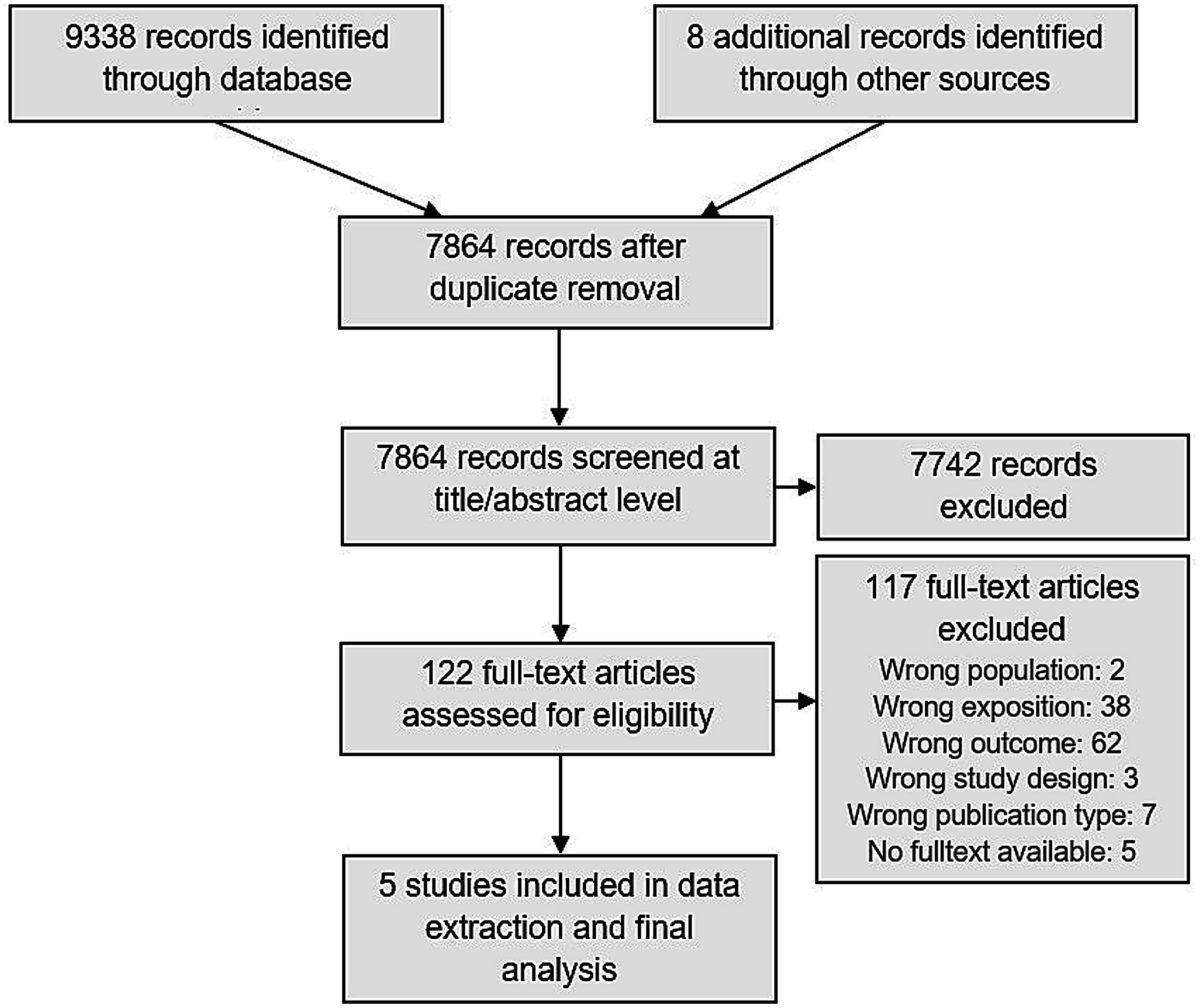
PRISMA flow chart (as suggested in 29–31).

The initial search identified 9,346 potential records. After removing duplicates, 7,864 articles were screened at the title and abstract level. Of these, 122 full-text articles were assessed for eligibility. In total, five studies were included in the final analysis (see PRISMA flow chart in Figure 1).

### 3.1 Characteristics of studies

Characteristics of each included study are summarized in Table 2. The five studies included were published between 2020 and 2024. As of data location, the studies have been conducted in the United Kingdom (37), India (26), the United States of America (38), Northern Cyprus (39), and South-Korea (40).

**Table 2:** Study characteristics.

Authors and publication year	Data location	Study design	Target group and number of participants	Demographics
Baungaard, Cathrine; Lane, Katie E.; Richardson, Lucinda (2023) (37)	United Kingdom	Mixed-methods; Cross-sectional	Nutrition students (final-year students in Association for Nutritionists (AfN) -accredited undergraduate nutrition courses); *n* = 51	87% female; 75% of participants between 18 to 25 years
Biswas, Aindrila (2020) (26)	India	Quantitative; Cross-sectional	Everybody in data collectors’ neighborhood; *n* = 247	n.a.
Bloyd Null, Dawn; Feeser, Kristiana; Kurtzhals, Geory (2023) (38)	USA (not clearly stated)	Quantitative; Cross-sectional	Undergraduates of a rural, medium-sized university; *n* = 339	63% female; Mean age = 19.2; Age range = 18–25
Kılıç, Hülya Fırat; Canatan, Sinem Dağ; Abiç, Arzu; Gök, Nur Demet (2024) (39)	Northern Cyprus	Quantitative; Cross-sectional	Nursing students; *n* = 316	65.5% female; Age range ≥ 18
Yoo, Hyelim; Jo, Eunbin; Lee, Hyeongyeong; Ko, Eunji; Jang, Eunjin; Sim, Jiwon; Kim, Kirang; Park, Sohyun (2023) (40)	South-Korea	Quantitative; Cross-sectional	Citizens; *n* = 4,039	51,9% female; Age range ≥18; Education attainments: n less than high school = 403 (10%), n high school graduates = 1,173 (29%), n more than college entrance = 2,463 (61%)

All studies were cross-sectional. Four of them used quantitative methods (26, 38–40) while one was designed as mixed-methods (37), although only the quantitative part provided relevant results for this review.

In three studies, students were the target group (37–39). The other two studies included citizens in general (26, 40). The sample size varried from 51 (37) to 4,039 (40).

Females constituted the larger group in the study sample in four out of five studies, making up between 51.9% (40) and 87% (37) of the sample. Biswas (2020) did not report on any sampling characteristics (26). Four studies did not provide information on the educational level of participants, whereas Yoo et al. (2023) records that most have more than a college entrance (40).

Only Biswas (2020) provided a conceptual model (“the literacy-sustainability-lifestyle-health modeling framework”) that was compiled by hypotheses and empirically tested (26).

### 3.2 Conceptual framework of environmental and sustainability-related literacy

All studies examined some form of environmental and sustainability-related literacy. Four studies focused specifically on environmental literacy (26, 37–39). In addition, Baungaard, Lane and Richardson assessed sustainable food literacy (37), while Biswas also investigated environmental attitude (26). Yoo et al. examined socio-ecological food literacy, thereby addressing a more specific subset of environmental literacy (40).

Although the understanding of environmental literacy was often similar and included translating information into behavior, the studies differed in what else they took into account when looking at literacy and what they presumed influences it. Similarly, the measurement tools varied with regard to the number of (sub-)dimensions assessed, the number of items included, and the scales applied (see Table 3).

**Table 3:** Understanding of environmental and sustainability-related literacy, health behaviors and corresponding measuring instruments in the included studies.

Authors and publication year	Environmental and sustainability-related literacy (exposition)		Health behaviors (outcome)	
	Understanding	Measuring instruments	Understanding	Measuring instruments
Baungaard, Lane and Richardson (2023) (37)	Environmental literacy: Ability to demonstrate environmentally conscious behavior Sustainable food literacy: Ability to make sustainable decisions regarding food	Environmental Literacy: Adapted from (70, 71); Included various facets of sustainable diet attitude, knowledge and literacy Sustainable food literacy: Adapted from (70–75); Examined knowledge questions about, e.g., food production and transport	Dietary intake: Shaped by individual (e.g., preferences, knowledge), sociocultural (e.g., beliefs, norms) and „food environment “factors (e.g., affordability, accessibility)	Adapted from (70, 71); Incorporated questions about frequency of consumption of different food groups, buying considerations (e.g., price and taste) and self-assessment of diet sustainability
Biswas (2020) (26)	Environmental Literacy: including knowledge, concern and awareness about the environment analyzing and processing information about the environment not just access to ecological education subjective approach including emotional and analytical skills influenced by cultural, socio-economic, political and environmental predecessor Environmental Attitude: includes expectation of outcomes of a certain behavior integrates social and psychological values, intents for behavior and beliefs	Environmental Literacy: Adapted and altered from (76); Included knowledge questions about environmental correlations and sustainable behavior in everday life Environmental Attitude: Adapted and altered from (77); Asked about the perceived future relevance of environmental conservation	Healthy lifestyle practices: self-centric affective and cognitive beliefs regarding the practice of a healthy lifestyle understanding of what is considered a healthy, pleasurable, and optimal lifestyle, characterized by low stress and the absence of harmful or physically detrimental behaviors determined by internal factors such as individual decisions, moral considerations, awareness and knowledge as well as external factors, e.g., economic or sociocultural influences	Adapted and altered from (78); Included healthy dietary practices, clean living environments and actively contributing to it
Bloyd Null, Feeser and Kurtzhals (2023) (38)	Environmental literacy: Extent to which people possess the ability to comprehend, interpret, and apply environmental information to make informed, sustainable, and environmentally responsible decisions	Environmental sustainability behaviors: Adapted from (79); Asked about time spent, e.g., recycling or saving water Sustainability knowledge/literacy via the Assessment of Sustainability Knowledge: Preexisting instrument from (80); Examined understanding of, e.g., sustainable living and pollution Sustainability attitudes via the Sustainability Attitudes Scale: Preexisting instrument from (80); Asked about attitudes about, e.g., consumerism and ecological challenges	Lifestyle: No further definition given by authors	Adapted from (79); Asked about, e.g., stress and sleep
Kılıç, Canatan, Abiç and Gök (2024) (39)	Environmental Literacy: Ability to transform environmental knowledge into behavior; fosters environmental awareness and sensitivity; facilitates learning natural laws thus encouraging relationship with nature	Environmental literacy scale: Preexisting instrument from (81); Assessed subdimensions environmental consciousness, environmental concern and environmental awareness Environmental Awareness and Sensitivity Scale: Preexisting instrument from (82); Included questions about, e.g., environmental behavior and interest in environmental issues	Smoking: No further definition given by authors	Origin unclear, probably part of „Descriptive Information Form“; Smoking habits: yes/no
Yoo et al. (2023) (40)	Socio-ecological Food Literacy: Ability to comprehend and apply diverse food-related information while taking into account the effects of dietary choices on society, the environment and health	Self-developed instrument; Assessed, e.g., ethical consumption, rural disparities and food sustainability	Food frequency: No further definition given by authors	Origin unclear; Examined average consumption frequency of various food groups, e.g., raw vegetables and whole grains but also fast food or sweetened beverages

### 3.3 Conceptual framework of health behaviors

Similar to the conceptual framework of environmental and sustainability-related literacy, the health behaviors considered as outcomes also varied across the studies, as well as the measurement tools employed (see Table 3).

Biswas and Bloyd Null, Feeser and Kurtzhals both used lifestyle-related measures, albeit with different operationalizations and instruments (26, 38). Biswas focused on healthy lifestyle practices, including, for example, healthy dietary practices (26), whereas Bloyd Null, Feeser and Kurtzhals examined aspects such as sleep and stress (38). Baungaard, Lane and Richardson and Yoo et al. assessed dietary intake and food frequency, respectively, as health behaviors, relying on different measurement approaches (37, 40). Kılıç et al. investigated smoking as a health behavior, measuring it through a binary (yes/no) self-report of smoking habits (39).

### 3.4 Quality appraisal

The quality of the studies is heterogeneous. Only criterion 3 of the JBI Critical Appraisal Checklist for cross-sectional studies, namely the validity and reliability of the exposure measurement, was consistently fulfilled across the studies (26, 37–40). Criteria 2, 7 and 8 (detailed description of study subjects and setting; validity and reliability of the outcome measurement; general consideration of appropriate statistical analysis) showed mixed findings across the studies. The remaining criteria 1, 5 and 6 (clarity of sample inclusion; identification of confounding factors; strategies to deal with confounding factors) were not adequately addressed in most of the studies. Criterion 4, which concerns the description of objective standard criteria for measuring the condition, was not applicable in any of the five studies, as they involved neither patients nor diagnostic methods. Notably, in the study of Yoo et al., all criteria except the unclear validity and reliability of the outcome measurement were rated positively (see Table 4) (40).

**Table 4:** Quality assessment of all included studies [modified from JBI critical appraisal checklist for cross-sectional studies (35, 36)].

JBI checklist for analytical cross-sectional studies	1. Clarity of sample inclusion	2. Detailed description of study subjects and setting	3. Validity and reliability of the exposure measurment	4. Description of the objective, standard criteria for measurement of the condition	5. Identification of confounding factors	6. Strategies to deal with confounding factors	7. Validity and reliability of the outcome measurement	8. Consideration of appropriate statistical analysis
Baungaard, Lane and Richardson (2023) (37)								
Biswas (2020) (26)								
Bloyd Null, Feeser and Kurtzhals (2023) (38)								
Kilic et al. (2024) (39)								
Yoo et al. (2023) (40)								

Yes = green; No = red; Unclear = yellow; N/A = gray.

### 3.5 Synthesis of results

A summary of the key findings of the studies is given in Table 5. Among the five studies, Biswas (2020) found a statistically significant influence of environmental and sustainability-related literacy on health behaviors based on a structural equation model approach and proposed a literacy-sustainability-lifestyle-health model. In this model, environmental literacy showed a statistically significant association on environmental attitude toward environmental conservation. Environmental attitude, in turn, facilitated healthy lifestyle practices. Moreover, environmental attitude also promoted sustainable lifestyle practices. Finally, healthy lifestyle practices were further explained through sustainable lifestyle practices (26).

**Table 5:** Summary of the key findings of the studies.

Authors and year	Relationship between environmental and sustainability-related literacy and health behaviors
Baungaard, Lane and Richardson (2023) (37)	Moderately strong negative correlation between sustainable food literacy and red meat intake (*r* = −0.445, *p* = 0.001) as well as poultry intake (*r* = −0.395, *p* = 0.004) No associations were reported for other food groups, nor between environmental literacy and dietary intake
Biswas (2020) (26)	Statistically significant influence of environmental literacy on healthy lifestyle practice Environmental literacy ➔ environmental attitude (ß = 0.32, *p* < 0.001); environmental attitude ➔ healthy lifestyle practices (ß = 0.51, *p* < 0.001) & sustainable lifestyle practices (ß = 0.46, *p* < 0.001); sustainable lifestyle practices ➔ healthy lifestyle practices (ß = 0.37, *p* < 0.001)
Bloyd Null, Feeser and Kurtzhals (2023) (38)	Statistically significant positive correlation between lifestyle and environmental sustainability behaviors (*r* = 0.395, *p* < 0.01) as well as sustainable attitudes (*r* = 0.241, *p* < 0.01) No statistically significant correlation between lifestyle and sustainable knowledge or literacy (*r* = −0.059)
Yoo et al. (2023) (40)	No information regarding the relationship between socio-ecological food literacy and food intake
Kılıç, Canatan, Abiç and Gök (2024) (39)	No statistically significant difference between students’ mean environmental literacy scores and smoking status (*p* = 0.281) Statistically significant positive association between environmental awareness and sensitivity scores among non-smokers (*p* = 0.003, *p* < 0.05)

✓, Association found. ×, No association found or no information reported.

The remaining four studies did not assess explanatory directions from environmental and sustainability-related literacy to health behaviors; instead, they identified a general cross-sectional association between the two domains.

In the study by Bloyd Null, Feeser, and Kurtzhals lifestyle—including health behaviors such as stress management, sleep, diet, and exercise—showed a statistically significant positive correlation with both environmental sustainability behaviors and sustainable attitudes. However, no statistically significant correlation was observed between lifestyle and sustainable knowledge or literacy (38).

The two following studies addressed food and dietary intake as health behavior. The study by Baungaard, Lane, and Richardson found a moderately strong negative correlation between sustainable food literacy and red meat intake as well as poultry intake, meaning that students who ate more red meat and poultry had lower sustainable food literacy scores. No associations were reported for other food groups, nor between environmental literacy and dietary intake (37).

In the study by Yoo et al. only results for the domain „nutrition and safety “were reported, as it showed the strongest association with food intake. No information was provided regarding socio-ecological food literacy (40).

The study by Kılıç, Canatan, Abiç, and Gök showed no statistically significant difference between students’ mean environmental literacy scores and smoking status. However, the environmental awareness and sensitivity scores were statistical significantly higher among non-smokers (39).

## 4 Discussion

The aim of this systematic review was to synthesize current findings on the association between environmental and sustainability-related literacy and health behaviors. We identified five cross-sectional studies that examined this association. Of them, the study of Biswas demonstrated an explanatory pathway for the influence of environmental and sustainability-related literacy on health behaviors (26), while three studies identified a general association between these two domains (37–39); one study did not report results for the specific association examined in this review (40). However, it should already be critically noted at this point that the model proposed by Biswas was based on a relatively small sample size (*n* = 247), which may limit the generalizability of the findings.

Overall, the findings suggest a positive relationship and highlight the potential that individuals with high environmental and sustainability-related literacy are more likely to engage in healthy behaviors. This is in line with previous studies examining thematically related constructs (e.g., the influence of environmental literacy on environmental behavior or health literacy on health-related behavior) that have generally reported positive associations (41–48). Beyond this overall association, a growing body of work has sought to identify the underlying mechanisms that translate literacy into behavior. For instance, a meta-analysis on health literacy found that self-efficacy and attitudes partially mediate its relationship with health behavior and outcomes (49), a pattern mirrored within this review by Biswas (2020), who showed that environmental attitude mediates the explanatory pathway from environmental literacy to both the sustainable and healthy lifestyle domains (26). These convergent empirical findings align closely with established attitude–behavior frameworks from behavioral science, such as the Theory of Planned Behavior, in which attitudes, perceived behavioral control, and self-efficacy translate knowledge into intention and, subsequently, behavior (50). They are also complemented by the environmental psychology literature on spillover and transfer effects, which suggests that engagement with one pro-environmental behavior may lead to the intention to engage in another pro-environmental behavior (51), as well as in the value-belief-norm (VBN) theory of environmentalism. The VBN theory proposes that pro-environmental behaviors emerge through a causal chain involving five variables: personal values (particularly altruistic values), beliefs (including the New Ecological Paradigm/ecological worldview, awareness of adverse consequences, and the ascription of personal responsibility), and personal norms for pro-environmental action (52, 53). Taken together, these perspectives suggest that environmental and sustainability-related literacy may not directly cause healthier behavior, but rather operate indirectly via shifts in attitudes, perceived norms, or self-efficacy. Yet, with the exception of Biswas (26), these pathways were not explicitly modeled in any of the included studies in this review. Embedding the findings within these broader theoretical frameworks therefore provides an important perspective for advancing the understanding of the mechanisms underlying health behaviors.

At the same time, it is important to note that the conceptualizations of environmental and sustainability-related literacy across the included studies in this review are highly heterogeneous. This interchangeable usage of terms and conceptual frameworks in the broader field of environmental and sustainability-related literacy is widespread, though not unproblematic, as it risks obscuring conceptual clarity (14, 15, 54). This highlights a key challenge for current research and indicates an urgent need for further research [see, e.g., (19)].

This conceptual heterogeneity is substantial rather than merely terminological: the included studies combine constructs that are theoretically distinct and operationalized differently, including environmental literacy (26, 37–39), sustainable food literacy (37), socio-ecological food literacy (40), environmental attitudes (26), and environmental awareness and sensitivity (39). While these constructs share a common emphasis on translating environmental knowledge into behavior, they differ in scope: environmental literacy is typically conceptualized as the broader, overarching construct (18), whereas sustainable food literacy and socio-ecological food literacy represent more narrowly defined, domain-specific applications focused on dietary and food-related contexts. It therefore remains unclear whether this review synthesizes evidence on a single coherent exposure construct or on multiple overlapping yet fundamentally different psychological and educational dimensions. A parallel concern applies to the outcome side: the health behaviors examined range from smoking status and sleep patterns to dietary intake, stress management, and broader lifestyle measures, spanning behavioral domains with distinct determinants, motivational pathways, and measurement traditions. This dual heterogeneity—on both the exposure and the outcome side—limits the interpretability of the synthesized findings and means that the reported positive relationship should be understood as a pattern observed across multiple related but non-identical construct pairings, rather than as evidence for a single, well-defined association.

It might also be assumed that considerably more studies have already examined this relationship, given its conceptual proximity to related fields such as environmental behavior or health literacy. However, despite a comprehensive and systematic search strategy applied across seven databases and supplemented by searches in Google Scholar, reference list screening, manual searches, and AI-based literature tools, no additional studies meeting our predefined eligibility criteria could be identified. While a broader body of literature has examined environmental behavior and health behavior, or environmental literacy and health literacy, as separate constructs (27, 28, 41–49), studies that specifically and empirically link environmental and sustainability-related literacy with health behaviors, as conceptualized in this review, remain scarce.

Nevertheless, the studies included in this review highlight important co-benefits linking sustainability and health, with clear implications for integrated climate and health interventions and policies that promote both environmental sustainability and human well-being. The findings underscore that sustainability and health are intertwined, since a healthy life is only possibly on a healthy planet (3, 20). Building on this, health promotion strategies targeting health-related behavior change should be combined with environmental and sustainability-related literacy—an approach that remains underutilized—to better translate knowledge into action, improve population health, and contribute to a healthy planet through sustainable health behaviors (20).

This optimistic framing, however, should be balanced against critical perspectives that are not fully captured by the included studies. Even where individuals possess high environmental and sustainability-related literacy, an intention-behavior gap may prevent this knowledge from translating into actual health-promoting behavior (55). Structural barriers, such as limited access to affordable healthy or sustainable food, inadequate active-travel infrastructure, or time and financial constraints, can also constrain behavior change regardless of individual literacy levels (56). Socioeconomic inequalities in access to environmental and sustainability-related education may further mean that literacy-focused approaches inadvertently widen rather than narrow health disparities (57). Moreover, health behaviors are shaped by political and commercial determinants, including food marketing, urban planning, and policy environments, which operate largely independently of individual literacy (58). For this reason, responsibility should not be placed solely on individuals; other stakeholders or so-called change agents such as policymakers or societal actors should also be held accountable (20, 59–61). Individual-level literacy interventions therefore have inherent limitations when addressing health and sustainability challenges that are embedded within broader systemic and structural crises. Future work should explicitly weigh literacy-focused approaches against these structural and political determinants, rather than treating individual knowledge and awareness as a sufficient lever for change. For the present review, however, it must be noted that this was not a focus of the included studies.

Our review has several limitations despite following a comprehensive search strategy that captured a wide range of studies across disciplines. Beyond the already mentioned challenges associated with the diverse environmental and sustainability-related literacy concepts as well as their measurement approaches, additional limitations should be acknowledged. First, we decided to include only studies that use the term “literacy” or “competencies” in combination with “sustainability,” “environmental” and related concepts. However, it is possible that additional relevant studies employ different terminology to represent such literacy concepts. Second, only five cross-sectional studies could be identified and based on their study design it was not possible to gain a deeper understanding of the underlying explanatory pathways. Third, the overall quality of the studies was relatively low, primarily due to methodological limitations such as small sample sizes, convenience sampling, limited generalizability, and adaptations of originally validated questionnaires. Notably, three of the five included studies were conducted among university student samples; this reliance on predominantly convenience-based student populations further constrains generalizability and should temper the broader claims regarding planetary health promotion and population-level interventions discussed above. Fourth, although the review protocol was preregistered with PROSPERO, some modifications were made after the commencement of the literature review. For instance, we intended to conduct a certainty assessment using the GRADE approach (62). But because of the low quality of the studies and their cross-sectional design, no GRADE assessment was conducted. Although the GRADE approach is widely regarded by international organizations as the gold standard for assessing certainty of evidence (63), it inherently downgrades evidence derived from non-randomized controlled trials (RCTs). As an alternative to a full GRADE assessment, we therefore provide a transparent narrative judgment of the overall certainty of evidence, following guidance for rating certainty in the absence of a pooled effect estimate (64): given the exclusively cross-sectional study designs, the small number of included studies, and the methodological limitations identified through the JBI appraisal, the overall certainty of evidence for the reviewed association between environmental and sustainability-related literacy and health behaviors should be considered low to very low. Finally, because all included studies relied exclusively on cross-sectional designs, the reported associations cannot be interpreted as evidence of causality or directionality—a limitation that applies to the evidence base as a whole and that we wish to emphasize explicitly. Reverse or bidirectional pathways, for example, individuals already engaged in healthier lifestyles subsequently seeking out environmental or sustainability-related information, remain equally plausible and cannot be ruled out based on the available evidence.

As the first systematic evidence synthesis of associations between environmental and sustainability-related literacy and health behaviors, our study yields several research and practical implications. We recommend the following key directions for research:

- Need for clear and consistent definitions and conceptual frameworks for different types of environmental and sustainability-related literacy [see, e.g., (19)].
- Development and/or use of robust and precise measurement instruments for each respective literacy concept, including the systematic reflection of its multiple dimensions (e.g., knowledge, skills, attitudes, and awareness) through appropriately designed items.
- Use of additional study designs beyond cross-sectional approaches, including, for example, the investigation of explanatory pathways, multiple measurement points and concrete interventions (e.g., longitudinal studies, randomized controlled trials [RCTs], or quasi-experimental designs).
- Investigation of underlying mechanisms and mediating or moderating factors (e.g., environmental attitudes, self-efficacy, socioeconomic status) linking environmental and sustainability-related literacy to health behaviors.
- Use of conceptual or behavior-change frameworks, or literacy-specific models to allow hypothesis-driven testing of underlying pathways.
- Use of qualitative research or mixed-methods studies to examine in depth the factors and determinants influencing literacy levels and their associations with health behaviors.
- Examination of potential bidirectional or reciprocal relationships, as health behaviors themselves may also influence engagement with environmental and sustainability-related literacy.
- Triangulation of self-reported measures with objective or behavioral indicators (e.g., accelerometry, dietary biomarkers, recorded waste or energy data) to reduce common-method bias and strengthen the validity of findings.
- Assessment of additional data from both high-income and low- and middle-income countries are required to draw robust conclusions and to better identify potential differences or commonalities.
- Investigation of differences between various target groups, such as men and women, to identify appropriate measures for enhancing environmental literacy in a targeted manner.
- Conduct of cross-cultural and cross-national comparative studies, as well as studies across different life stages (e.g., children, older adults) and vulnerable populations, to examine the generalizability of findings across diverse contexts.
- Examination of education and training programs aimed at increasing environmental and sustainability-related literacy as interventions, including, for example, the evaluation of their outcomes as well as their effective design and implementation.

Practical implications to enhance environmental and sustainability-related literacy and to promote actions that improve environmental conditions and health behaviors lie, for example, in educational interventions from primary school onward, as well as environmental sustainability-oriented marketing and communication campaigns in schools and universities (38, 39). For instance, curricula can be refined by integrating environmental competencies and literacy as content areas, while simultaneously strengthening these competencies through appropriate teaching methods and learning activities (e.g., recycling, sustainable diets, energy conservation) alongside health education elements (37). Furthermore, physical and organizational changes in the environments of schools and universities (e.g., accessible recycling facilities or plogging) can raise awareness and increase recycling behaviors as well as physical activity on campuses (38, 65).

## 5 Conclusion

This systematic review provides initial indications of associations between environmental and sustainability-related literacy and health behaviors. Findings suggest that individuals with higher levels of environmental and sustainability-related literacy tend to exhibit better health behaviors (26, 37–39).

Overall, the existing body of research in this area is limited, and further studies—especially those employing longitudinal designs—are needed to better understand causal relationships between environmental and sustainability-related literacy and health behaviors. Addressing the conceptual heterogeneity surrounding environmental and sustainability-related literacy is essential so that information about complex human–environment relationships can be accessed, understood, critically reflected upon, and collectively translated into sustainable, healthy, and transformative action.

However, responsibility should not rest solely with individuals; policymakers and other societal actors must also be held accountable, as addressing environmental challenges ultimately requires collective action (20, 26, 59–61, 66). This calls for systems-based and Health in All Policies approaches (20, 67–69). In this context, health promoters could play a key coordinating role, which in turn requires a reconceptualization of their competencies and responsibilities, positioning them as central actors in advancing planetary health promotion (20).

## Data Availability

The original contributions presented in the study are included in the article/Supplementary material, further inquiries can be directed to the corresponding author.
