# Supplementary material for: Relationship between environmental and sustainability-related literacy and health behaviors: a systematic review
Source: Front Public Health. 2026 Jul 27;14:1860395. doi: 10.3389/fpubh.2026.1860395 (PMC13454104; doi:10.3389/fpubh.2026.1860395)
Supplement: Supplementary file 1 [file Data_Sheet_1.PDF]

Search strategies

Search run for each database on 9 April 2025.

Database	Search term
**PubMed/MEDLINE**<br><br>6,310 hits	(((literacy[TIAB] OR competenc*[TIAB])<br><br>AND (sustainab*[TIAB] OR planet*[TIAB] OR environmental*[TIAB] OR climate*[TIAB] OR eco[TIAB] OR ecolog*[TIAB] OR eco?friendly[TIAB] OR eco?conscious[TIAB] OR earth*[TIAB] OR transformati*[TIAB] OR green[TIAB] OR renewable[TIAB] OR natur*[TIAB] OR carbon[TIAB] OR biodiversity*[TIAB]))<br><br>AND<br><br>(((live[TIAB] OR living[TIAB] OR life*[TIAB] OR behav*[TIAB] OR acti*[TIAB]) AND (health*[TIAB] OR unhealth*[TIAB] OR prevent*[TIAB] OR physical*[TIAB] OR sedentary[TIAB])) OR movement[TIAB] OR sport*[TIAB] OR exercise*[TIAB] OR walk*[TIAB] OR “physical inactiv*”[TIAB] OR gardening[TIAB] OR cycl*[TIAB] OR bicycl*[TIAB] OR biking[TIAB] OR „active transport“ [TIAB] OR „active travel“ [TIAB] OR „active mob*“[TIAB] OR „active commuting“ [TIAB] OR<br><br>((health*[TIAB] OR unhealth*[TIAB]) AND (diet*[TIAB] OR nutrition*[TIAB] OR food[TIAB] OR eat?[TIAB] OR snack*[TIAB] OR cook*[TIAB] OR drink*[TIAB])) OR<br><br>((mindful*[TIAB] OR resilien*[TIAB]) AND (acti*[TIAB] OR behav*[TIAB] OR life*[TIAB] OR live[TIAB])) OR<br><br>smok*[TIAB] OR vap?[TIAB] OR overdrinking[TIAB] OR ((use? [TIAB] OR using[TIAB] OR abus*[TIAB] OR consum*[TIAB]) AND (cigarette*[TIAB] OR tobacco[TIAB] OR vape*[TIAB] OR alcohol*[TIAB] OR substance*[TIAB] OR drug*[TIAB])) OR<br><br>screen?time[TIAB] OR<br><br>sleep*[TIAB]))
**OVID/MEDLINE**<br><br>605 hits	((((literacy or competenc*) adj3 (sustainab* or ecolog* or climate* or planet* or eco?friendly or eco or eco?conscious or environmental* or earth* or transformati* or green or natur* or carbon or renewable* or biodiversity*)) or eco?literacy) and (((live or living or life* or behav* or acti*) adj3 (health* or unhealth* or prevent* or physical* or sedentary)) or movement or sport* or exercise* or walk* or (physical adj1 inactiv*) or gardening or cycl* or bicycl* or biking or (active adj (transport or travel or mob* or commuting)) or ((health* or unhealth*) adj3 (diet* or nutrition* or food or eat* or snack* or cook* or

	drink*)) or ((mindful* or resilien*) adj3 (acti* or behav* or life* or live)) or smok* or vap* or overdrinking or ((use* or using or abus* or consum*) adj (cigarette* or tobacco or vape* or alcohol* or substance* or drug*)) or screen?time or sleep*)).ti,ab.
**Scopus**<br>1,250 hits	TITLE-ABS-KEY((((literacy OR competenc*) W/3 (sustainab* OR ecolog* OR climate* OR planet* OR eco?friendly OR eco OR eco?conscious OR environmental* OR earth* OR transformati* OR green OR natur* OR carbon OR renewable* OR biodiversity*)) OR eco?literacy) AND (((live OR living OR life* OR behav* OR acti*) W/3 (health* OR unhealth* OR prevent* OR physical* OR sedentary)) OR movement OR sport* OR exercise* OR walk* OR (physical PRE/1 inactiv*) OR gardening OR cycl* OR bicycl* OR biking OR (active PRE/1 (transport OR travel OR mob* OR commuting)) OR ((health* OR unhealth*) W/3 (diet* OR nutrition* OR food OR eat* OR snack* OR cook* OR drink*)) OR ((mindful* OR resilien*) W/3 (acti* OR behav* OR life* OR live)) OR smok* OR vap* OR overdrinking OR ((use* OR using OR abus* OR consum*) PRE/1 (cigarette* OR tobacco OR vape* OR alcohol* OR substance* OR drug*)) OR screen?time OR sleep*))
**Web of Science (WoS)**<br>723 hits	(((literacy OR competenc*) NEAR/3 (sustainab* OR ecolog* OR climate* OR planet* OR eco$friendly OR eco OR eco$conscious OR environmental* OR earth* OR transformati* OR green OR natur* OR carbon OR renewable* OR biodiversity*)) OR eco$literacy) AND (((live OR living OR life* OR behav* OR acti*) NEAR/3 (health* OR unhealth* OR prevent* OR physical* OR sedentary)) OR movement OR sport* OR exercise* OR walk* OR (physical NEAR/1 inactiv*) OR gardening OR cycl* OR bicycl* OR biking OR (active NEAR/1 (transport OR travel OR mob* OR commuting)) OR ((health* OR unhealth*) NEAR/3 (diet* OR nutrition* OR food OR eat* OR snack* OR cook* OR drink*)) OR ((mindful* OR resilien*) NEAR/3 (acti* OR behav* OR life* OR live)) OR smok* OR vap* OR overprinting OR ((use* OR using OR abus* OR consum*) NEAR/1 (cigarette* OR tobacco OR vape* OR alcohol* OR substance* OR drug*)) OR screen$time OR sleep*)
**APA PsycINFO (via Ebscohost)**<br>283 hits	(((literacy OR competenc*) N3 (sustainab* OR ecolog* OR climate* OR planet* OR eco#friendly OR eco OR eco#conscious OR environmental* OR earth* OR transformati* OR green OR natur* OR carbon OR renewable* OR biodiversity*)) OR eco#literacy)<br>AND<br>(((live OR living OR life* OR behav* OR acti*) N3 (health* OR unhealth* OR prevent* OR physical* OR sedentary)) OR movement OR sport* OR exercise* OR walk* OR physical N1 inactiv* OR gardening OR cycl* OR bicycl* OR biking OR (active N1 (transport OR travel OR mob* OR commuting)) OR<br>((health* OR unhealth*) N3 (diet* OR nutrition* OR food OR eat* OR snack* OR cook* OR drink*)) OR

	((mindful* OR resilien*) N3 (acti* OR behav* OR life* OR live)) OR<br><br>smok* OR vap* OR overdrinking OR ((use* OR using OR abus* OR consum*) N1 (cigarette* OR tobacco OR vape* OR alcohol* OR substance* OR drug*)) OR<br><br>screen#time OR<br><br>sleep*)
**PSYNDEX Literature with PSYNDEX Tests (via Ebscohost)**<br><br>19 hits	(((literacy OR competenc*) N3 (sustainab* OR ecolog* OR climate* OR planet* OR eco#friendly OR eco OR eco#conscious OR environmental* OR earth* OR transformati* OR green OR natur* OR carbon OR renewable* OR biodiversity*)) OR eco#literacy)<br><br>AND<br><br>(((live OR living OR life* OR behav* OR acti*) N3 (health* OR unhealth* OR prevent* OR physical* OR sedentary)) OR movement OR sport* OR exercise* OR walk* OR physical N1 inactiv* OR gardening OR cycl* OR bicycl* OR biking OR (active N1 (transport OR travel OR mob* OR commuting)) OR<br><br>((health* OR unhealth*) N3 (diet* OR nutrition* OR food OR eat* OR snack* OR cook* OR drink*)) OR<br><br>((mindful* OR resilien*) N3 (acti* OR behav* OR life* OR live)) OR<br><br>smok* OR vap* OR overdrinking OR ((use* OR using OR abus* OR consum*) N1 (cigarette* OR tobacco OR vape* OR alcohol* OR substance* OR drug*)) OR<br><br>screen#time OR<br><br>sleep*)
**GreenFILE (via Ebscohost)**<br><br>148 hits	(((literacy OR competenc*) N3 (sustainab* OR ecolog* OR climate* OR planet* OR eco#friendly OR eco OR eco#conscious OR environmental* OR earth* OR transformati* OR green OR natur* OR carbon OR renewable* OR biodiversity*)) OR eco#literacy)<br><br>AND

	(((live OR living OR life* OR behav* OR acti*) N3 (health* OR unhealth* OR prevent* OR physical* OR sedentary)) OR movement OR sport* OR exercise* OR walk* OR physical N1 inactiv* OR gardening OR cycl* OR bicycl* OR biking OR (active N1 (transport OR travel OR mob* OR commuting)) OR<br><br>((health* OR unhealth*) N3 (diet* OR nutrition* OR food OR eat* OR snack* OR cook* OR drink*)) OR<br><br>((mindful* OR resilien*) N3 (acti* OR behav* OR life* OR live)) OR<br><br>smok* OR vap* OR overdrinking OR ((use* OR using OR abus* OR consum*) N1 (cigarette* OR tobacco OR vape* OR alcohol* OR substance* OR drug*)) OR<br><br>screen#time OR<br><br>sleep*)
**Total hits**	**9,338**
